# A retrospective observational study of clinical presentation and management outcomes in patients with paroxysmal supraventricular tachycardia at a tertiary center in Bahrain

**DOI:** 10.3389/fcvm.2026.1739540

**Published:** 2026-04-14

**Authors:** Osama Zeidan, Aljazi Alohaly, Ali Zayer, Noor Jaragh, Maryam Alqayem, Ayat Ziedan, Salim Fredericks, Walid Assar

**Affiliations:** 1 School of Medicine, Royal College of Surgeons in Ireland, Medical University of Bahrain (RCSI-MUB), Busaiteen, Bahrain; 2 College of Medicine, Sultan Qaboos University, Muscat, Oman; 3 Cardiology Department, King Hamad University Hospital, Muharraq, Bahrain

**Keywords:** adenosine, cardioversion, comorbidities, emergency department, paroxysmal supraventricular tachycardia, PSVT, vagal maneuvers

## Abstract

**Objectives:**

This study aimed to evaluate the clinical characteristics, symptom presentation, and management outcomes of patients diagnosed with paroxysmal supraventricular tachycardia (PSVT) at King Hamad University Hospital (KHUH), a tertiary care center. It examined the influence of demographics, comorbidities, and sex on treatment responses to pharmacologic and non-pharmacologic interventions, including cardioversion.

**Methods:**

A retrospective observational study was conducted on 427 adult patients with PSVT (ICD-10: I47.1, confirmed by chart review) between January 2018 and early 2024. Data were extracted from the KHUH HOPE electronic medical records system. Descriptive statistics summarized baseline characteristics; chi-square tests examined categorical associations. Kaplan–Meier analyses for the cardioversion subgroup (*n* = 18) are presented as exploratory and descriptive only.

**Results:**

Females comprised 56.9% of the cohort (mean age 52.8 ± 15.2 years). Palpitations were the most common symptom (94.4%). Of 219 patients who received adenosine, 177 (80.8%) achieved cardioversion with the first dose, 25 (11.4%) with the second, and 17 (7.8%) with the third. Vagal maneuvers showed reduced success in patients with structural heart disease and diabetes mellitus. Electrical cardioversion was required in 18 patients (4.2%). Pacemaker implantation was documented in 3 patients (0.7%) for co-existing conduction disease, not as a PSVT treatment. Comorbidities significantly influenced outcomes: hypertension was associated with favorable vagal maneuver response, while heart failure and ischemic heart disease correlated with poor response across all modalities.

**Conclusion:**

PSVT generally responds well to first-line treatments. However, individualized strategies are warranted for patients with structural heart disease or significant comorbidities. Observed sex-based and age-related response differences are descriptive and require multivariable validation.

## 1 Introduction

Paroxysmal supraventricular tachycardia (PSVT) is a supraventricular arrhythmia characterized by episodic, abrupt-onset and termination of regular tachycardia. It affects individuals across all age groups and is frequently encountered in emergency departments (EDs), with presentations ranging from asymptomatic episodes to severe manifestations such as chest pain, dizziness, or syncope (1). Electrocardiographically, PSVT is typically identified by a narrow QRS complex, though P waves may not be clearly discernible (2). While classic symptoms include palpitations, dyspnea, chest discomfort, and syncope, atypical presentations such as burping, chills, tinnitus, and absence-like seizures have also been reported (3).

PSVT also places a significant burden on emergency departments through frequent revisits (31). These diverse presentations can mimic psychiatric or gastrointestinal disorders and may delay diagnosis (3). Although the estimated prevalence of PSVT is 2.25 per 1,000 individuals, the actual burden is likely underreported (4). PSVT encompasses AVNRT, AVRT, and focal atrial tachycardia (4). Though not life-threatening, its recurrent symptoms significantly impair quality of life, and it contributes to over 50,000 ED visits annually in the United States (5, 6, 21).

Treatment begins with vagal maneuvers, followed by adenosine (6 mg initial dose, up to 12 mg), and in refractory cases, cardioversion or ablation (2, 7, 22). Age and sex influence subtype distribution and treatment response: AVRT predominates in younger males, while AVNRT and AT are more prevalent in older females (1, 8). Despite extensive literature on PSVT management in Western populations, data from the Gulf region remain scarce. This study addresses that gap and contributes locally representative data to guide regional clinical practice (9, 23).

## 2 Materials and methods

### 2.1 Study design and setting

This is a retrospective observational study conducted at King Hamad University Hospital (KHUH), a tertiary care centre in Bahrain. The primary aim was to describe clinical presentation, treatment patterns, and cross-sectional associations between patient characteristics and treatment outcomes. Kaplan–Meier analyses are explicitly presented as exploratory and descriptive only, without inferential conclusions. Data were anonymized per the Institutional Research and Ethics Committee at KHUH (IRB approval: 2024-812).

### 2.2 Study population

**Figure 1:**
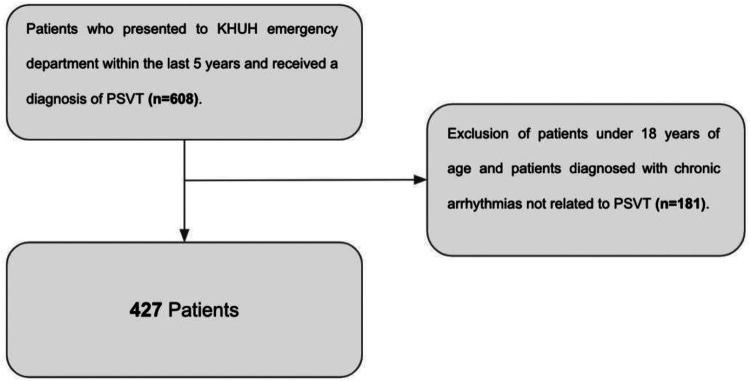
Study flowchart: inclusion and exclusion criteria. Of 608 patients initially identified by ICD-10 code I47.1, 181 were excluded (age <18 years or chronic arrhythmias not consistent with PSVT), yielding a final cohort of 427 patients.

Adult patients (≥18 years) presenting to the KHUH ED between January 2018 and early 2024 with a PSVT diagnosis (ICD-10 code I47.1) were included. ICD-10 identification was supplemented by chart review to verify ECG findings consistent with narrow-complex regular tachycardia and to exclude documented atrial fibrillation, atrial flutter, or other chronic supraventricular arrhythmias. Systematic subtype differentiation (AVNRT/AVRT/AT) was not consistently available—a recognized limitation. Of 608 initially identified patients, 427 met eligibility criteria (Figure 1).

### 2.3 Data collection and variables

Data were extracted from the HOPE EMR by five independent researchers and entered into a secure Microsoft Excel spreadsheet. Variables included demographics, presenting symptoms, comorbidities, management strategies, laboratory parameters, and treatment outcomes (Table 1).

**Table 1:** Summary of extracted variables From electronic medical record.

Category	Variables
Demographics	Age, sex, nationality
Clinical Data	Presenting symptoms, comorbidities (hypertension, diabetes, IHD, thyroid disease), family history
Management	Vagal maneuvers (type), adenosine (dose/number), cardioversion, pacemaker implantation
Laboratory Data	Electrolytes, renal function, hemoglobin, WBC, cardiac biomarkers
Outcomes	Treatment response (sinus rhythm restoration), recurrence, need for escalation

### 2.4 Statistical analysis

All analyses used IBM SPSS v23. Categorical variables are reported as frequencies and percentages within their respective subgroup denominators. Continuous variables are expressed as mean ± SD or median (IQR). Comparisons used chi-square tests; *p* < 0.05 was significant (two-tailed). Due to incomplete capture of key confounders (hemodynamic status, tachycardia duration, prior therapy), multivariable logistic regression was not performed—acknowledged as a limitation. Kaplan–Meier analyses (*n* = 18) are descriptive only; no log-rank comparisons are made.

## 3 Results

### 3.1 Baseline characteristics

Of 427 patients, 243 (56.9%) were female and 184 (43.1%) male; mean age was 52.8 ± 15.2 years. Most patients (83.4%) were Bahraini nationals. Palpitations were most common (94.4%), followed by chest pain (33.7%), shortness of breath (23.9%), dizziness (19.0%), and sweating (8.0%). Syncope and weakness each occurred in 3.7%; 0.9% were asymptomatic. Hypertension was the most prevalent comorbidity (38.1%), followed by diabetes mellitus (27.8%), thyroid disease (13.8%), ischemic heart disease (5.8%), and heart failure (2.3%); 33.2% had no comorbidities. Baseline characteristics are summarized in Table 2.

**Table 2:** Baseline characteristics of the study population (*n* = 427).

Baseline Characteristics of the Study Population	
**Variable**	Count (%)
**Gender**	
Male	184 (43.1%)
Female	243 (56.9%)
**Nationality**	
National (Bahraini)	356 (83.4%)
(Non-Bahraini)	71 (16.6%)
**Age (years)**	
18–29	49 (11.47%)
30–39	29 (6.79%)
40–49	82 (19.20%)
50–59	133 (31.16%)
≥60	134 (31.39%)
**Comorbidities**	
Hypertension (HT)	163 (38.1%)
Diabetes Mellitus (DM)	119 (27.8%)
Thyroid Disease	59 (13.8%)
Ischemic Heart Disease (IHD)	25 (5.8%)
Heart Failure (HF)	10 (2.3%)
no comorbidities	142 (33.2%)

### 3.2 Treatment outcomes by Sex and Age group

**Figure 2:**
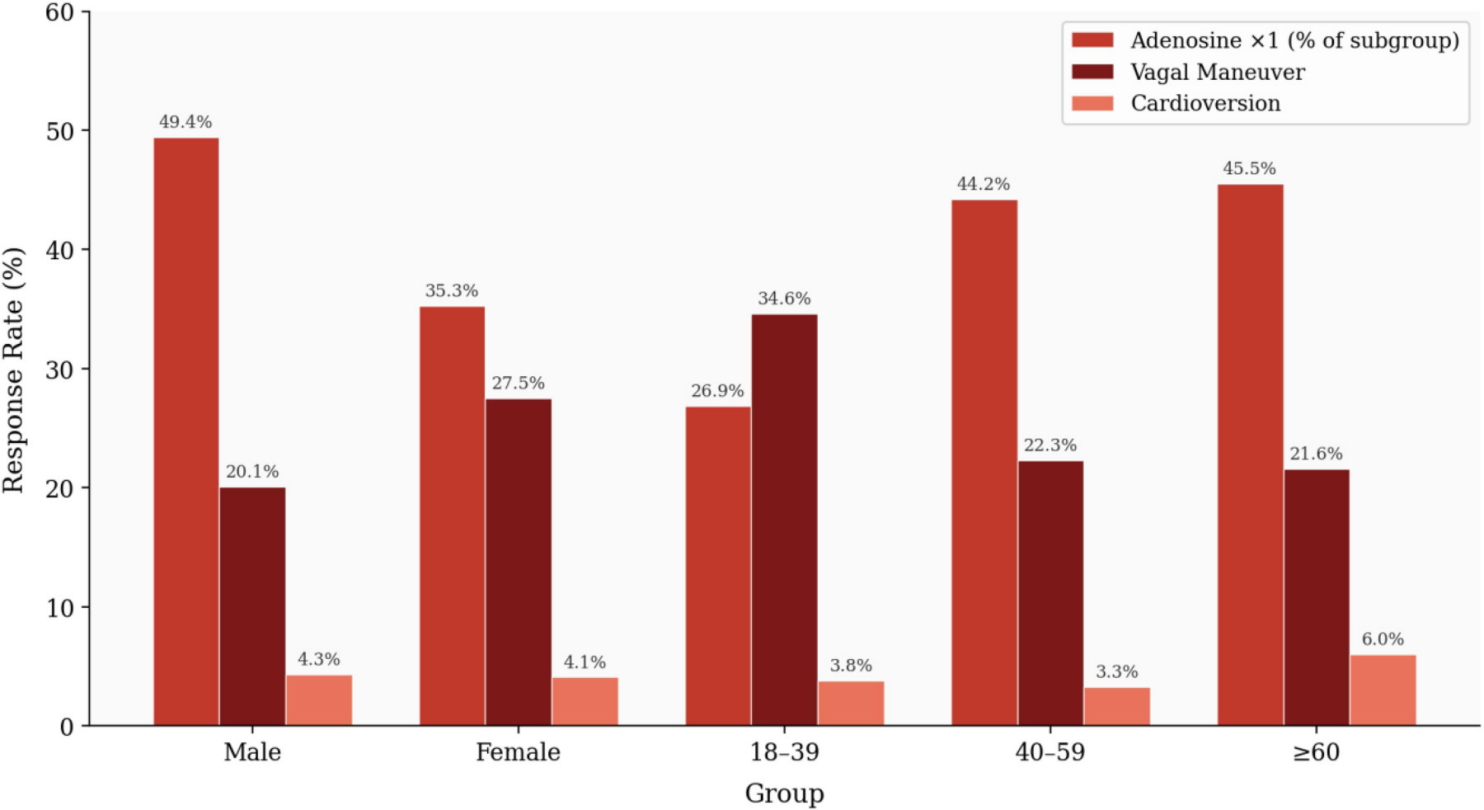
Treatment response by Sex and Age group in PSVT. Percentages represent proportions within each subgroup. All three treatment modalities showed statistically significant variation by sex (vagal: *p* = 0.048; adenosine × 1: *p* = 0.002) and by age group (vagal: *p* = 0.03; adenosine × 1: *p* = 0.003).

Spontaneous resolution occurred in 42 patients (9.8%). Of 219 adenosine-treated patients, 177 achieved cardioversion after the first dose (80.8% of those treated; 41.5% of total cohort), 25 required a second dose (11.4% of those treated), and 17 a third (7.8% of those treated). Vagal maneuvers were attempted in 156 patients; carotid sinus massage was effective alone in 44 (28.2% of those receiving it), and other vagal techniques in 104 (66.7%). Electrical cardioversion was required in 18 patients (4.2%). Pacemaker implantation was documented in 3 patients (0.7%)—all for co-existing sinus node or AV conduction disease, not acute PSVT treatment (see Section 4.4). Treatment outcomes by sex and age are shown in Table 3 and Figure 2.

**Table 3:** Treatment effectiveness by Sex and Age group (% within each subgroup; n in parentheses = subgroup size).

Group	Spont.	Carotid Massage	Vagal Mnvr	Aden. x1	Aden. x2	Aden. x3	Cardio-version	p
All (*n* = 427)	42 (9.8%)	44 (10.3%)	104 (24.4%)	177 (41.5%)	25 (5.9%)	17 (4.0%)	18 (4.2%)	<0.001
Male (*n* = 184)	17 (9.2%)	19 (10.3%)	37 (20.1%)	91 (49.4%)	7 (3.8%)	5 (2.7%)	8 (4.3%)	<0.001
Female (*n* = 243)	25 (10.2%)	25 (10.2%)	67 (27.5%)	86 (35.3%)	18 (7.4%)	12 (4.9%)	10 (4.1%)	<0.001
p (M vs F)	0.72	0.99	0.048^a^	0.002^a^	0.049^a^	0.16	0.92	
Age 18–39 (*n* = 78)	9 (11.5%)	7 (9.0%)	27 (34.6%)	21 (26.9%)	6 (7.7%)	5 (6.4%)	3 (3.8%)	<0.001
Age 40–59 (*n* = 215)	20 (9.3%)	25 (11.6%)	48 (22.3%)	95 (44.2%)	11 (5.1%)	9 (4.2%)	7 (3.3%)	<0.001
Age ≥60 (*n* = 134)	13 (9.7%)	12 (9.0%)	29 (21.6%)	61 (45.5%)	8 (6.0%)	3 (2.2%)	8 (6.0%)	<0.001
p (age)	0.85	0.76	0.03^a^	0.003^a^	0.63	0.22	0.50	

^a^ *p* < 0.05. Percentages are proportions within each subgroup.

Females demonstrated higher vagal maneuver success (27.5% vs. 20.1%; *p* = 0.048) while males had higher first-dose adenosine success (49.4% vs. 35.3%; *p* = 0.002). Patients aged 40–59 had the highest adenosine first-dose success (44.2%; *p* = 0.003); those aged 18–39 had the highest vagal maneuver success (34.6%). Patients ≥60 required cardioversion most frequently (6.0%). These are descriptive associations and may reflect unmeasured confounders.

### 3.3 Comorbidities and their impact on treatment

The impact of comorbidities on treatment outcomes is summarized in Table 4. Hypertensive patients (*n* = 163) had moderate vagal maneuver success (20.9%) and 37.4% adenosine first-dose conversion within this subgroup. Diabetic patients (*n* = 119) had the lowest vagal maneuver success (10.9%) but the highest adenosine first-dose success (58.8%). Thyroid disease patients (*n* = 59) showed notably higher vagal maneuver success (35.6%). Ischemic heart disease patients (*n* = 25) had no spontaneous resolution; adenosine was effective in 28% at first dose. Heart failure patients (*n* = 10) had the poorest overall response. Patients without comorbidities had the highest adenosine first-dose success (51.4%).

**Table 4:** Comorbidities and their impact on treatment (% within each comorbidity subgroup).

Comorbidity	Spont.	Carotid Massage	Vagal Mnvr	Aden. x1	Aden. x2	Aden. x3	Cardio-version	p
Hypertension (*n* = 163)	21 (12.9%)	19 (11.7%)	34 (20.9%)	61 (37.4%)	18 (11.0%)	4 (2.5%)	6 (3.7%)	<0.001
Diabetes (*n* = 119)	8 (6.7%)	7 (5.9%)	13 (10.9%)	70 (58.8%)	6 (5.0%)	8 (6.7%)	7 (5.9%)	<0.001
Thyroid Dis. (*n* = 59)	3 (5.1%)	1 (1.7%)	21 (35.6%)	26 (44.1%)	3 (5.1%)	1 (1.7%)	4 (6.8%)	<0.001
IHD (*n* = 25)	0 (0%)	1 (4.0%)	8 (32.0%)	7 (28.0%)	3 (12.0%)	5 (20.0%)	1 (4.0%)	0.002
Heart Failure (*n* = 10)	0 (0%)	1 (10.0%)	2 (20.0%)	5 (50.0%)	1 (10.0%)	0 (0%)	1 (10.0%)	0.21
No comorbidities (*n* = 142)	14 (9.9%)	16 (11.3%)	29 (20.4%)	73 (51.4%)	6 (4.2%)	2 (1.4%)	2 (1.4%)	<0.001

IHD, Ischemic heart disease. Percentages are proportions within each comorbidity subgroup.

### 3.4 Pharmacological management: adenosine stepwise response

**Figure 3:**
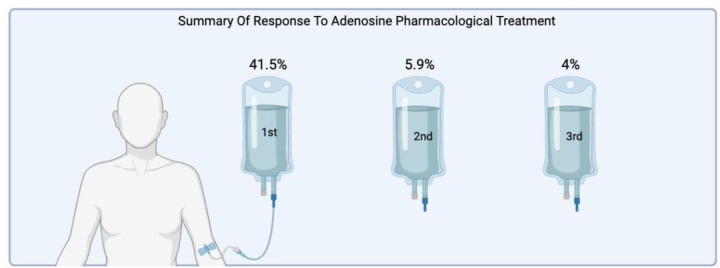
Stepwise response to adenosine therapy in patients With PSVT. Proportions shown represent percentages of all 427 patients (41.5%, 5.9%, 4.0%). Among the 219 patients who actually received adenosine, first-, second-, and third-dose success rates were 80.8%, 11.4%, and 7.8%, respectively. Figure created with BioRender.com.

Figure 3 illustrates the stepwise response to adenosine therapy. Of 219 patients who received adenosine, 80.8% responded to the first dose (6 mg), 11.4% to the second (12 mg), and 7.8% to the third. These proportions reflect treatment efficacy among those who actually received the drug.

### 3.5 Exploratory survival analysis following cardioversion

**Figure 4:**
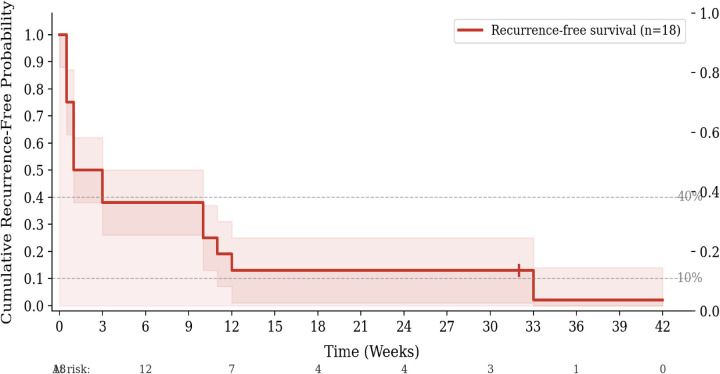
Exploratory Kaplan–Meier analysis: cumulative recurrence-free probability following cardioversion (*n* = 18). Shaded band represents approximate 95% confidence interval. + denotes censored observations. These data are descriptive only and should not be used for inferential purposes given the small sample size and absence of systematic prospective follow-up.

**Figure 5:**
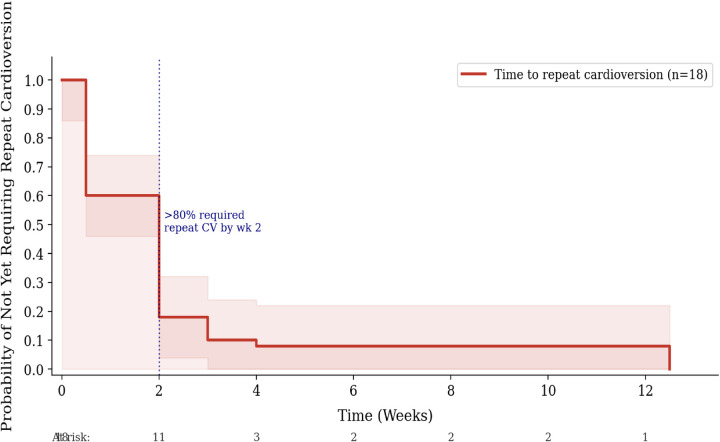
Exploratory Kaplan–Meier analysis: time to repeat cardioversion after SVT recurrence (*n* = 18). Over 80% of patients who recurred required repeat cardioversion within 2 weeks (dotted blue line). Shaded band = approximate 95% CI. Descriptive only.

Among the 18 patients who underwent electrical cardioversion, Kaplan–Meier analysis showed the cumulative recurrence-free probability fell below 40% by week 3 and approached 10% by week 12 (Figure 4). Among those who recurred, over 80% required repeat cardioversion within 2 weeks (Figure 5). These analyses are presented as preliminary and exploratory given the small sample (*n* = 18) and retrospective follow-up; no inferential conclusions are drawn. The observed short recurrence-free intervals highlight a potentially high-risk subgroup warranting early electrophysiology referral.

## 4 Discussion

### 4.1 Baseline characteristics and demographics

This study describes the demographic and clinical profile of PSVT in a Middle Eastern population. The observed female predominance (56.9%) is consistent with published literature linking AVNRT—the most common PSVT subtype—to higher rates in women, likely reflecting autonomic and electrophysiological differences (10). The mean age of 52.8 years aligns with prior findings that PSVT typically presents in middle-aged individuals, with AVRT more common in younger males and AVNRT in older females (1). Although subtype differentiation was not systematically available in this dataset, the demographic patterns are consistent with these epidemiological trends.

Concurrent infection was present in 8% of patients, supporting the role of systemic inflammation as an arrhythmogenic stressor (11). Electrolyte values were within normal ranges; however, wide variability in creatinine levels may reflect underlying renal dysfunction, a recognized arrhythmia risk factor (12). The predominantly Bahraini cohort (83.4%) highlights the value of locally representative data for guiding regional clinical decision-making.

### 4.2 Pharmacological management

Adenosine was the most commonly used pharmacologic agent. When expressed as a proportion of the 219 patients who received it, first-dose cardioversion was achieved in 80.8%—consistent with controlled-setting success rates of 80%–95% (13). The 41.5% figure represents the proportion of the full cohort and is presented for context; both are reported transparently. Sex-based differences in adenosine response may reflect pharmacokinetics, receptor density differences, or comorbidity distribution, but causal attribution is not possible from these observational data (14). Figure 3 visually summarizes the stepwise response pattern.

### 4.3 Non-Pharmacological approaches

**Figure 6:**
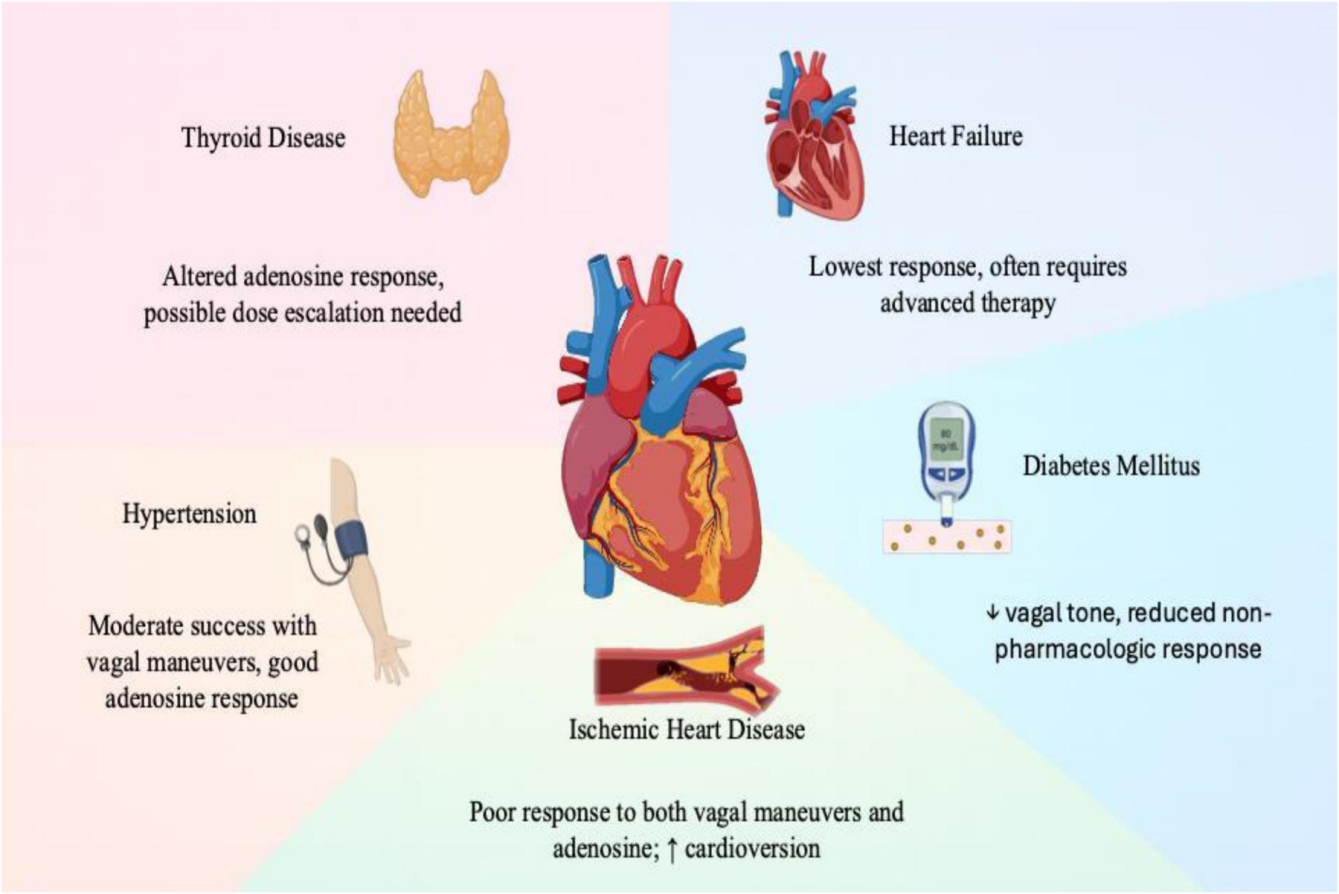
Comorbidity-Specific response patterns to PSVT interventions. Each quadrant summarizes the dominant treatment response pattern for the indicated comorbidity. IHD, ischemic heart disease. Figure created with BioRender.com.

Vagal maneuvers showed moderate overall success, with better outcomes in patients with preserved autonomic function (26, 33). Diabetic patients had markedly reduced vagal maneuver success (10.9%), consistent with the effects of diabetic autonomic neuropathy on parasympathetic tone (5, 30). Patients with thyroid disease showed higher vagal maneuver success (35.6%), possibly reflecting altered AV nodal excitability (15). Structural heart disease patients (IHD, heart failure) had the poorest response across all modalities, consistent with impaired conduction and autonomic dysregulation (16, 17). Figure 2 visually summarizes treatment response by sex and age group, and Figure 6 illustrates comorbidity-specific response patterns. Structural heart disease patients had the poorest response across all modalities, consistent with impaired conduction and autonomic dysregulation (16, 17, 32).

### 4.4 Pacemaker implantation

Three patients (0.7%) had a documented record of pacemaker implantation. It must be explicitly noted that pacemaker implantation is not a treatment for PSVT. In all three cases, the implantation was attributable to sinus node dysfunction or AV conduction block—conditions that may co-exist with or develop independently of PSVT. These cases are reported descriptively only and should not be interpreted as a PSVT treatment outcome.

### 4.5 Sex-Based response patterns

Observed sex differences in treatment response—higher vagal maneuver success in females and higher first-dose adenosine success in males—may partly reflect differences in PSVT subtype distribution, comorbidity profiles, and treatment selection rather than direct hormonal or receptor-level mechanisms (18, 24). While estrogen has been proposed in some experimental studies to modulate AV nodal conduction (18, 27), direct inference from this cross-sectional observational dataset is not warranted. Multivariable analyses accounting for age, comorbidity burden, and PSVT subtype are needed to clarify these relationships (25).

### 4.6 Comorbidities and clinical implications

Comorbidities substantially influenced PSVT management outcomes (Figure 6). Hypertension was associated with relatively preserved baroreceptor function and moderate vagal success. Diabetic patients—likely due to autonomic neuropathy—showed the lowest vagal maneuver success but good adenosine response. Structural heart disease (IHD, heart failure) was associated with the poorest outcomes across all modalities, necessitating earlier cardioversion. Thyroid disease patients showed altered adenosine pharmacodynamics but good vagal response. These findings support early comorbidity-stratified escalation pathways in the emergency setting (16, 17, 19, 20).

### 4.7 Strengths and limitations

Strengths include a relatively large single-center cohort (*n* = 427) from an underrepresented region, systematic data extraction by five independent researchers, and comprehensive comorbidity profiling.

Key limitations are: (1) Retrospective design—treatment assignment was not randomized and may reflect unmeasured clinical judgment. (2) Diagnostic accuracy—while ICD-10 coding was supplemented by chart review, formal ECG-based rhythm classification into AVNRT/AVRT/AT subtypes was not consistently available, limiting mechanistic inference and increasing misclassification risk. (3) Missing clinical confounders—blood pressure at presentation, hemodynamic instability, tachycardia duration, prior antiarrhythmic therapy, and electrophysiology study/ablation history were not systematically captured, precluding multivariable adjustment. (4) No multivariable analysis—reported associations may be confounded by comorbidity clustering, age distribution, and treatment selection bias. (5) Small cardioversion subgroup (*n* = 18)—Kaplan–Meier analyses lack statistical power and should be considered hypothesis-generating only. (6) Single-center data from one region of Bahrain—PSVT treated elsewhere was not captured, potentially underestimating recurrence rates (28).

## 5 Conclusion

This study provides one of the first large-scale analyses of PSVT management in a Middle Eastern population. While adenosine and vagal maneuvers remain effective first-line therapies, treatment response varies with sex, age, and comorbidity burden. Patients with ischemic heart disease or heart failure showed consistently poor outcomes, often requiring cardioversion. These findings support a risk-stratified, individualized approach to PSVT management (29). The descriptive survival data following cardioversion suggest a high-risk subgroup warranting structured post-discharge follow-up and early specialist referral.

Formal causal conclusions are not warranted without multivariable adjustment. Future multicenter, prospective studies with systematic ECG-based rhythm classification, comprehensive clinical data capture, and standardized follow-up are needed to validate these findings and guide individualized PSVT care in the Gulf region.

## Data Availability

The raw data supporting the conclusions of this article will be made available by the authors, without undue reservation.
